# Aneurysmal bone cyst of the rib: a case report

**DOI:** 10.4076/1752-1947-3-8457

**Published:** 2009-09-09

**Authors:** Murat Yasaroglu, Bulend Ketenci, Hatice Demirbag, Mehmet Yildirim, Ilgaz Dogusoy

**Affiliations:** 1 Siyami Ersek Thoracic and Cardiovascular Surgery Center, Istanbul, Turkey

## Abstract

**Introduction:**

An aneurysmal bone cyst is a benign, but expansile tumor like lesion that generally occurs in the long bones including the vertebral column. An aneurysmal bone cyst arising from the rib, especially in the elderly, is extremely rare.

**Case presentation:**

We report a 58-year-old Turkish woman with an aneurysmal bone cyst of the right 3rd rib treated with chest wall resection. The pathologic findings confirmed the diagnosis of aneurysmal bone cyst. The patient has been followed up for 5 years with no evidence of recurrence.

**Conclusion:**

En bloc resection can be curative and provide good results for this rare type of chest wall tumor.

## Introduction

Aneurysmal bone cyst (ABC) was first reported by Jaffe and colleagues in 1942. They used this term to describe the "blow out" radiographic appearance and blood filled contents of the cystic spaces [1]. ABC is a non-neoplastic bone tumor that occurs predominantly in children and young adults, mainly involving the long bones and the vertebrae [2]. ABC occurring as a primary rib tumor is unusual, especially in the elderly [3]-[5]. We report a case of ABC of the right 3rd rib treated with chest wall resection.

## Case presentation

A 58-year-old Turkish woman was admitted to our hospital complaining of pain from a swelling on the right side of her chest for the previous 4 years. She had no history of trauma and her past medical history was unremarkable. Physical examination revealed a 10 cm × 7 cm tender palpable mass in the midaxillary line with otherwise normal findings.

**Figure 1:**
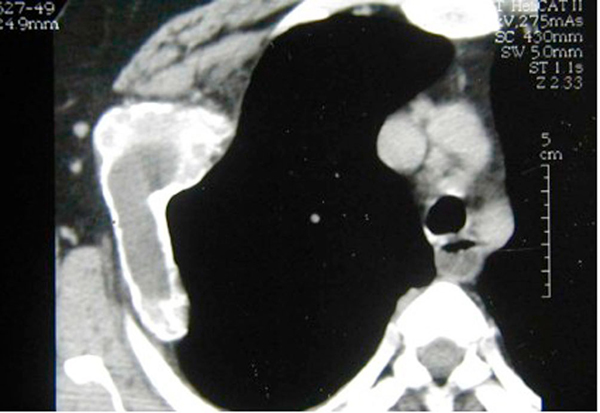
**Computed tomography of the lesion showed a multiloculated, expanding mass with fluid levels in the right 3rd rib**.

Her routine laboratory test results were normal. Posterior-anterior (PA) chest X-ray demonstrated a purely lytic expansile lesion originating from the right 3rd rib with ballooning of the cortex. Computed tomography (CT) of the lesion showed a multiloculated, expanding mass with fluid levels in the right 3rd rib. The mass was protruding into the hemithorax and the adjacent soft tissue (Figure 1). The cortex overlying it was intact. A radionuclide bone scan showed increased uptake at the site of the lesion and no other abnormalities. Fine needle aspiration cytology (FNAC) demonstrated hemosiderin laden macrophages and multinucleated osteoclastic giant cells. Through a right posterolateral thoracotomy, the lesion was explored and found to be localized in the anterior three-quarters of the 3rd rib with multiple cystic components, extending into the surrounding intercostal muscles. The visceral pleura were intact. The cystic mass in the 3rd rib and the adjacent segments of the 2nd and 4th ribs were resected along with the intercostal muscles. The chest wall was reconstructed with a double layered Prolene mesh (Ethicon, Inc., Somerville, NJ, USA). The postoperative period was uneventful and the patient was discharged on the eighth day following the surgery.

**Figure 2:**
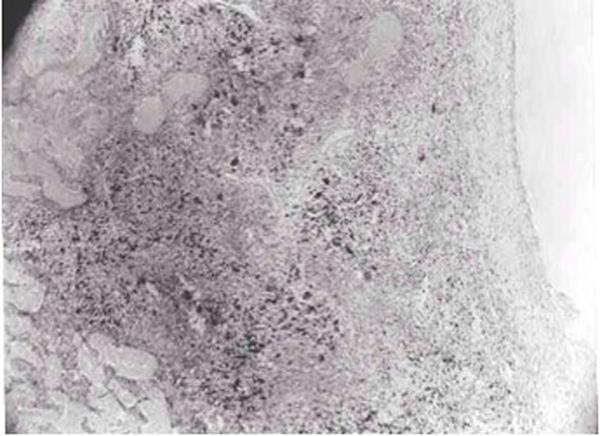
**Reactive bone trabeculae, immature connective tissue and granulation tissue containing fibroblasts, myofibroblasts and hemosiderotic macrophages (×40, hematoxylin and eosin stain)**.

The microscopic examination demonstrated blood filled multiple cavernous spaces separated by fibrous connective tissue, with hemosiderin pigment laden macrophages; multinucleated osteoclastic giant cells and well-developed vascular spaces with hemorrhages in the septa lining the cystic spaces, confirming the diagnosis of ABC (Figure 2).

**Figure 3:**
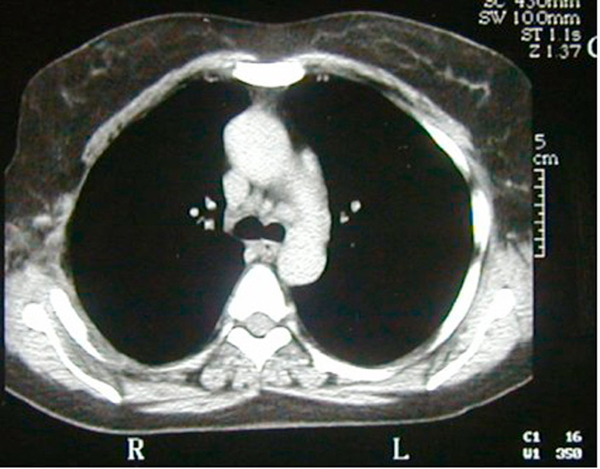
**Computed tomography scan of the patient 5 years after the surgery with no evidence of recurrence**.

Our patient has been followed up for 5 years with no evidence of recurrence or functional disturbance (Figure 3).

## Discussion

Primary rib neoplasms are uncommon. They comprise 5% to 7% of all primary bone tumors [2,4]. ABC presenting as a primary rib tumor is very rare. It is a benign, progressive tumor that comprises 1.3% of all primary bone tumors [5]. It may involve any bone, mostly the spine and the long bones but localization in the ribs is unusual [2,3]. ABC has been observed in every rib except the lower three [6]. The age of our patient is unusual for ABC since it is extremely rare in the elderly. Approximately 80% of ABC occurs in patients who are younger than age 20. There is no race or sex predominance [2].

ABC is not a true cyst or aneurysm [3]. The etiology is unknown but circulatory disturbance due to arteriovenous malformation is widely accepted [2,5,6]. Grossly, the lesion consists of a paper-thin cortex and multiple blood filled cavities. Microscopically, the patient had a primary ABC with no additional bone tumor. The lesion should be designated as a primary ABC if it has a uniform histological pattern or as a secondary ABC if the lesion contains another bone tumor such as fibrous dysplasia, non-ossifying fibroma, osteoblastoma or chondromyxoid fibroma [2]. The patient was symptomatic with complaints of pain and swelling. About 29% of reported cases have been found incidentally by a routine chest X-ray. Dyspnea, paraplegia and pathologic fractures are less frequent symptoms [5].

The typical radiographic appearance of ABC is that of an eccentric, lytic, "blow out" type of lesion, demarcated by a thin shell of subperiosteal new bone [1,6]. There is often expansion into the adjacent soft tissues. Computed tomography and magnetic resonance imaging (MRI) are useful in the diagnosis of ABC. We evaluated the patient with CT scans. The appearance of multiple cavernous spaces filled with fluid levels on CT scans was suggestive of ABC. The expansion of ABC into the adjacent intercostal muscle indicated an aggressive stage. However, it should be emphasized that the radiographic appearance is not sufficiently specific to establish a definitive diagnosis. Radiological differential diagnosis should include: giant cell tumor, plasmocytoma, chondromyxoid fibroma, chondrosarcoma, fibrous dysplasia, and metastasis [5].

Once the lesion is diagnosed, treatment should be initiated as soon as possible since local extension and rapid growth can cause pathologic fractures, paralysis due to spinal cord compression, compression of the vital organs and malignant transformation [4,6]. The current methods of treatment include curettage bone grafting, curettage and cryosurgery, radiation, or en bloc resection [2]. The treatment of choice is complete excision of the rib involvement of ABC. Other treatment modalities result in a higher recurrence rate [6]. FNAC and radiological evaluation suggested a benign pathology in our patient. The lesion was resected along with adjacent ribs to avoid recurrence. Complete resection offers the best chance for cure in both benign and malignant lesions of the chest wall. Incomplete resection of ABC may result in rapid recurrence [6,7]. Reconstruction with double-layered Prolene mesh (Ethicon) provides good functional results. No further treatment was required in our patient and she was healthy with no recurrence observed after 5 years of follow up.

## Conclusion

ABC is a rare, benign pathology, which should be considered in the differential diagnosis of chest wall tumors. Complete surgical excision can be the best treatment for cure.

## Consent

Written informed consent was obtained from the patient for publication of this case report and any accompanying images. A copy of the written consent is available for review by the Editor-in-Chief of this journal.

## Competing interests

The authors declare that they have no competing interests.

## Authors' contributions

MY and HD performed the operation. BK was the major contributor in writing the manuscript. MY performed the histological examination of the mass. ID was the departmental chair.
